# Characterization of Umami Compounds and Volatile Profiles of Honeybee Brood Umami Powder Under Optimized Drying Conditions: Implications for Sensory Properties

**DOI:** 10.3390/foods15122234

**Published:** 2026-06-20

**Authors:** Supakit Chaipoot, Sirinthip Jaijoi, Gochakorn Kanthakat, Kuntathee Chaimueng, Chalermkwan Somjai, Pairote Wiriyacharee, Rajnibhas Sukeaw Samakradhamrongthai, Pattavara Pathomrungsiyounggul, Worachai Wongwatcharayothin, Rewat Phongphisutthinant

**Affiliations:** 1 Multidisciplinary Research Institute, Chiang Mai University, Chiang Mai 50200, Thailand; ninesrt1991@gmail.com (S.J.); kuntathee.c@gmail.com (K.C.); 2 Center of Excellence in Microbial Diversity and Sustainable Utilization, Chiang Mai University, Chiang Mai 50200, Thailand; 3 Faculty of Agro-Industry, Chiang Mai University, Chiang Mai 50100, Thailand; gochakorn.g@gmail.com (G.K.); rajnibhas.s@cmu.ac.th (R.S.S.); pattavara.p@cmu.ac.th (P.P.); 4 Processing and Product Development Factory, The Royal Project Foundation, Chiang Mai 50100, Thailand; chalermkwansomjai@hotmail.com (C.S.); pairote.w@cmu.ac.th (P.W.); 5 Faculty of Humanities, Chiang Mai University, Chiang Mai 50200, Thailand; worachai.w@cmu.ac.th

**Keywords:** honeybee brood, umami compound, 5′-nucleotides, sensory evaluation, volatile flavor compounds

## Abstract

Honeybee brood is a nutrient-rich food source containing natural umami-active compounds, such as glutamic acid, aspartic acid, and 5′-nucleotides, which are responsible for its characteristic umami taste. This study aimed to optimize drying conditions to enhance the umami composition and sensory properties of honeybee brood umami powder (HBb-UP). A factorial design was employed to evaluate the effects of drying temperature and time on umami-related amino acids, 5′-nucleotides, and equivalent umami concentration (EUC). Drying temperature and time significantly influenced the formation of umami compounds, with the optimized drying condition (65 °C for 3 h) promoting higher umami composition and improved sensory attributes of HBb-UP. Volatile flavor analysis using GC–MS and an electronic nose revealed a diverse range of aroma compounds contributing to the overall flavor profile. Descriptive sensory evaluation and electronic tongue analysis indicated that umami and saltiness were the dominant taste attributes, accompanied by mild seasoning and fishy notes associated with interactions between amino acids and nucleotides. Principal component analysis demonstrated positive correlations among umami-related amino acids, nucleotides, EUC, and sensory attributes, confirming their combined contribution to taste perception. These findings highlight the potential of optimized HBb-UP as a natural flavor enhancer and functional ingredient for use in sustainable food systems.

## 1. Introduction

In recent years, edible insects have attracted increasing global attention, driven by the urgent need for sustainable, nutritious, and environmentally friendly food sources. With the global population rising and growing concerns about food shortages and the environmental impact of traditional livestock farming, edible insects are increasingly recognized as a sustainable alternative source of protein [1]. Among the diverse array of insect-based foods, honeybee brood, consisting of the larval and pupal stages of honeybees, has attracted increasing attention due to its high nutritional value and culinary potential [2]. Although traditionally consumed in several tropical and subtropical regions, honeybee brood remains underutilized and has received comparatively limited scientific and industrial attention as a mainstream food ingredient [3]. Honeybee brood is characterized by its high-quality protein, essential amino acids, beneficial fatty acids, vitamins, and minerals, which have attracted increasing interest in its development as a functional and sustainable food ingredient [4,5,6]. Honeybee brood also has a relatively low ecological footprint compared to conventional livestock, positioning it as a promising alternative protein source that aligns with current trends toward environmentally sustainable and circular food systems [4]. Its nutritional value and favorable physicochemical properties support its use in dietary supplements, bakery products, and meal replacements [6]. Furthermore, the presence of taste-active compounds contributes to its inherent umami characteristics, enabling its potential use as a natural flavor enhancer [7,8].

The nutritional richness of honeybee brood, particularly its protein and bioactive compound content, has supported growing interest in its use as a sustainable food resource. It contains a diverse range of flavor-active constituents, particularly free amino acids such as glutamic acid (137 mg/g protein) and aspartic acid (81 mg/g protein), which are predominant contributors to umami taste perception, along with alanine, which imparts sweetness and mild savory notes [9]. Haber et al. [10] reported that honeybee brood powder obtained through freeze-drying is a nutritionally valuable product, containing 20–25% protein (dry matter basis), as well as high levels of antioxidant activity and polyphenols. Sensory evaluation further indicated that its aroma profile is predominantly characterized by buttery and milky attributes. In addition, 5′-nucleotides, including inosine monophosphate (IMP), guanosine monophosphate (GMP), and adenosine monophosphate (AMP), may also be present as nitrogen-containing compounds that contribute to overall taste characteristics. Beyond its chemical composition, honeybee brood is closely associated with biological functions related to taste perception in honeybees. Studies on honeybee gustatory perception indicate that their taste receptors respond to essential nutrients derived from pollen sources, particularly amino acids and related compounds. Furthermore, honeybees, especially egg-laying females, exhibit a preference for sugar solutions enriched with amino acids [11]. These compositional and biological characteristics highlight the importance of processing strategies in modulating the flavor profile and functional properties of honeybee brood-derived products.

Thermal treatment represents an important processing stage in the preparation of honeybee brood powder, as it influences both flavor development and nutritional quality through mechanisms such as protein degradation, amino acid release, and non-enzymatic reactions. Controlled heating can enhance savory attributes via reactions such as the Maillard reaction and the release of amino acids from proteins while also contributing to microbial safety. In addition, thermal processing may enhance certain bioactivities, including antioxidant activity, and increase the availability of amino acids and bioactive peptides. It may also promote the formation of compounds associated with prebiotic functionality [12,13,14]. Collectively, these observations support the sensory relevance of amino acid-related taste compounds in honeybee-derived materials. Although honeybee brood has been recognized for its nutritional composition and potential umami characteristics, the effects of thermal drying conditions on flavor development have remained insufficiently explored. Moreover, the relationships among umami compounds, volatile profiles, and sensory properties have not yet been comprehensively investigated. Therefore, this study aimed to optimize drying conditions for the production of honeybee brood-derived umami powder and to comprehensively characterize its umami compounds, volatile profiles, and sensory properties. Furthermore, the relationships among chemical composition, flavor profiles, and sensory attributes were evaluated to provide insights into the mechanisms underlying umami taste perception.

## 2. Materials and Methods

### 2.1. Materials and Chemical Reagents

Honeybee brood (*Apis mellifera* L.) was obtained from a Longan honey farm located in Mae-wang District, Chiang Mai Province, Thailand. The raw honeybee brood, which had already died prior to processing, was steamed in a stainless-steel steamer under atmospheric pressure (~100 °C) for 1 h and then stored in plastic bags at −20 °C until further analysis. This steaming step was applied uniformly to all samples as a standardized pre-treatment to ensure sample consistency and stabilization prior to drying optimization. The chemicals, reagents, and standards used in this study included ethanol, hexane, and methanol (AR grade) (RCI Labscan, Bangkok, Thailand); N-acetyl-L-cysteine (Merck, Darmstadt, Germany); O-phthalaldehyde (OPA) (Sigma-Aldrich, Tokyo, Japan); potassium sulfate, sodium citrate tribasic dihydrate, and sodium hydroxide (RCI Labscan, Bangkok, Thailand); and sodium carbonate (QRec, Auckland, New Zealand). Furthermore, 17 amino acid reference standards obtained from Wako Pure Chemical Co., Osaka, Japan, were used for amino acid analysis. The 5′-nucleotide standards (5′-GMP, 5′-IMP, and 5′-XMP) were purchased from ChemCruz™ Biochemicals (Santa Cruz Biotechnology, Inc., Dallas, TX, USA).

### 2.2. Preparation and Drying Optimization of Honeybee Brood for Umami Powder Production

Honeybee brood samples were prepared and dried in a hot-air oven (UNE600, Memmert, Schwabach, Germany) following previously reported methods [13,14]. The drying process was investigated and optimized using a factorial design, in which temperature and drying time were evaluated within the ranges of 50–80 °C and 3–24 h, respectively. A 2^2^ factorial design (two factors: drying temperature and drying time) with three center-point replicates was employed, and the experimental conditions are summarized in Table 1. Optimal drying conditions were determined based on physicochemical properties, umami-related compounds, and sensory evaluation. Then, samples dried under the optimal conditions were ground using a blender (Stormmix Co., Ltd., Samut Prakan, Thailand). For extract preparation, 100 g of ground D-HBb was mixed with 500 mL of deionized water (1:5, *w*/*v*). The mixture was heated at 95 °C for 10 min in a water bath to facilitate the extraction of water-soluble umami compounds, followed by sterilization in an autoclave at 109 °C for 16 min to standardize preparation of the honeybee brood umami solution prior to foam-mat drying and ensure microbial safety. The extract was passed through a 10 µm nylon filter bag to separate the honeybee brood umami solution, which was then processed using foam-mat drying to produce HBb-UP.

For honeybee brood umami powder (HBb-UP) production, the umami solution was processed according to Chaipoot et al. [12] using optimized levels of key variables, including honeybee brood extract, glycerol monostearate (GMS), and carboxymethyl cellulose (CMC), under foam-mat drying technology.

All experimental runs were evaluated for physicochemical properties, umami composition (moisture content, color values, water activity (aw), 5′-nucleotide compounds, equivalent umami concentration, and amino acid profile) and sensory evaluation. These evaluations were used to determine the effects of the experimental processing conditions using Design-Expert Version 12.0 (Stat-Ease Inc., Minneapolis, MN, USA).

### 2.3. Physicochemical Properties and Umami Composition of Honeybee Brood

#### 2.3.1. Color Values Analysis

The honeybee brood color characteristics (L*, a*, b*) were assessed using a CR-400 colorimeter (Konica Minolta, Tokyo, Japan), where L* represents lightness, a* represents the red–green component, and b* represents the yellow–blue component.

#### 2.3.2. Water Activity (aw) and Moisture Content

The aw value was assessed at 25 °C with a three-channel water activity meter (model AWC200, Novasina, Lachen, Switzerland), following the procedure previously reported by Chaipoot et al. [12]. Moisture content was determined according to AOAC method 925.10 [15]. All measurements were performed in triplicate.

#### 2.3.3. Amino Acids Profile Analysis

Seventeen amino acids were determined in the sample using a post-column derivatization method with a Shim-pack Amino-Na column, following the Shimadzu protocol and the method described by Somjai et al. [16]. The amino acids analyzed included aspartic acid, threonine, serine, glutamic acid, proline, glycine, alanine, cysteine, valine, methionine, isoleucine, leucine, tyrosine, phenylalanine, histidine, lysine, and arginine. A Shim-pack Amino-Na column (100 mm × 6.0 mm i.d., 5 µm; P/N: 228-18837-91, Shimadzu, Kyoto, Japan) coupled with a Prominence RF-20A fluorescence detector (Shimadzu, Kyoto, Japan) was used. Mobile phases A, B, and C, consisting of sodium citrate buffers with pH values of 3.23 (A) and 10.0 (B), and an aqueous solution of 0.2 M NaOH (C), were employed. For the pre-column derivatization of amino acids, reaction reagents were prepared using ortho-phthalaldehyde (OPA) and N-acetyl-L-cysteine. The operating conditions included a flow rate of 0.4 mL/min, a column oven temperature of 60 °C, and an injection volume of 10 µL. Amino acid concentrations were determined for all samples in triplicate.

#### 2.3.4. 5′-Nucleotide Compounds and Equivalent Umami Concentration (EUC) Analysis

HPLC analysis of three 5′-nucleotide compounds (5′-GMP, 5′-IMP, and 5′-XMP) was performed according to the method described by Harada-Padermo et al. [17], with slight modifications. A Shimadzu HPLC system, featuring an Inertsil ODS-3 column (250 mm × 4.6 mm, 5 µm particle size, GL Sciences Inc., Tokyo, Japan) was used in conjunction with a photodiode array detector set to operate at a wavelength of 254 nm. Compound elution was performed using a gradient method with two mobile phases: mobile phase A, a 50 mM potassium phosphate buffer adjusted to pH 4.8, and mobile phase B, absolute methanol (HPLC grade). The gradient program was as follows: 0–5 min, 0% B; 5–14 min, linear increase to 10% B; 14–22.5 min, 10% B; 23–30 min, 0% B. The column temperature was maintained at 30 °C, with a flow rate of 0.5 mL/min and an injection volume of 10 µL. Each sample was analyzed in triplicate.

The EUC represents the umami taste intensity resulting from the combined contribution of monosodium glutamate (MSG)-like amino acids, such as aspartic acid and glutamic acid, together with umami-related 5′-nucleotides [18]. The EUC was determined by calculating the concentrations of umami-related amino acids (glutamic acid and aspartic acid) and 5′-nucleotides (5′-GMP, 5′-IMP, and 5′-XMP) using Equation (1), as proposed by Chaipoot et al. [19].

EUC (g MSG/100 g) = Ʃ a_i_b_i_ +1218 × (Ʃ a_i_b_i_) × (Ʃ a_j_b_j_) (1)

where EUC represents the umami taste intensity, expressed in grams of monosodium glutamate (MSG) per 100 g of sample; ai indicates the concentration (g/100 g) of glutamic acid and aspartic acid; a_j_ represents the concentration (g/100 g) of 5′-nucleotides; b_i_ corresponds to the relative umami concentration for each amino acid (glutamic acid = 1, aspartic acid = 0.077); b_j_ signifies the relative umami concentration for 5′-nucleotides (5′-IMP = 1, 5′-GMP = 2.3, 5′-XMP = 0.61); and 1218 denotes the synergistic constant based on the concentration used (g/100 g).

### 2.4. Sensory Evaluation and Descriptive Analysis (DA)

A preliminary sensory evaluation of 10 experimental runs was conducted using a 9-point hedonic scale following Evans et al. [7]. A total of 25 student participants (*n* = 25), comprising master’s and PhD students from the Department of Agro-Industry, Chiang Mai University, assessed HBb-UP based on sensory attributes including appearance, color, aroma, and overall acceptance. The 9-point hedonic evaluation was used to preliminarily assess consumer acceptance of appearance, aroma, and overall acceptance under different drying conditions. These sensory responses were included in the optimization process together with physicochemical properties and umami-related compounds to support the selection of suitable drying conditions, rather than to serve as analytical sensory measurements. As the panel consisted of untrained participants, the hedonic data were interpreted as indicators of product acceptability rather than sensory intensity.

To further elucidate the sensory characteristics of optimized HBb-UP, descriptive analysis (DA) was conducted to generate a comprehensive sensory profile. A trained panel consisting of 10 assessors was selected from graduate students in the Department of Agro-Industry, Chiang Mai University, based on their sensory acuity and prior experience in sensory evaluation. Panelists underwent training sessions to develop and standardize sensory terminology, during which consensus was reached on attribute definitions and reference standards. The attribute definitions and reference standards established during panel training are provided in Table S1. The final attribute set included appearance, color intensity, odor and flavor (e.g., salty, protein, fishy, insect, umami, sweet), and aftertaste (oily, sweet). Samples were identified using random three-digit codes and served in randomized order under controlled environmental conditions (25–27 °C, white lighting). Panelists evaluated each attribute using unstructured 15 cm line scales anchored with “low intensity” and “high intensity,” and the scores were converted to numerical values (1–15). Each sample was evaluated in duplicate, and panelists were instructed to cleanse their palate with water between samples to minimize carryover effects. The collected data were used to quantify the intensity of sensory attributes and to construct the sensory profile of optimized HBb-UP (Supplementary Material File).

### 2.5. Instrumental Analysis of Flavor and Taste Compounds in Optimized HBb-Up

#### 2.5.1. Flavor Profiling Analysis Using Gas Chromatograph–Electronic Nose (GC–E-Nose)

A modified analytical procedure based on the method reported by Haber et al. [10] was employed. Samples were pre-incubated at 60 °C for 5 min prior to injection. A 5 mL volume of headspace gas was injected at a rate of 125 µL/s, with the injector maintained at 200 °C. The trap temperature was held at 40 °C, with a split flow rate of 10 mL/min. The column temperature was initially set to 50 °C, ramped to 80 °C at 1 °C/s, then further increased to 250 °C at 3 °C/s, and held at 250 °C for 21 s. The detector temperature was maintained at 260 °C.

#### 2.5.2. Taste Profile Analysis Using Electronic Tongue (E-Tongue)

Taste profiles were analyzed using an Astree electronic tongue (Alpha M.O.S., Toulouse, France) equipped with a #6 sensor array comprising seven sensors. Among these, AHS, CTS, NMS, ANS, and SCS are cross-sensitive sensors commonly associated with sourness, saltiness, umami, sweetness, and bitterness, respectively, whereas PSK and CPS function as reference sensors for signal stabilization and calibration. The electronic tongue mimics human taste perception by using cross-sensitive sensors to detect dissolved compounds and generate electrical response patterns, which are interpreted as taste-related fingerprints for comparative flavor characterization [20]. Following each measurement, the sensors were cleaned with deionized water to prevent carryover effects. Sensor responses recorded during the stable signal period (100–120 s) were averaged and used as the representative output value. All samples were evaluated in triplicate.

#### 2.5.3. Volatile Compounds Analysis Using Gas Chromatography–Mass Spectrometry (GC–MS)

Volatile compounds were extracted using a 50/30 μm DVB/CAR/PDMS SPME fiber at 37 °C for 10 min. The extracted compounds were then analyzed using a 7890A gas chromatograph coupled with a 5975C mass spectrometer (Agilent Technologies, Santa Clara, CA, USA), following the method described by Haber et al. [10]. Separation was carried out on a DB-5MS fused silica capillary column (5%-phenyl-methylpolysiloxane; 30 m × 0.25 mm i.d., 0.25 μm film thickness). Samples were injected at a split ratio of 1:10 at 250 °C, with helium (purity 99.999%) as the carrier gas at a constant flow rate of 1.0 mL/min. The oven temperature program was as follows: initial temperature of 50 °C (held for 5 min), ramped at 5 °C/min to 200 °C with no hold, and then increased at 10 °C/min to 220 °C and held for 5 min. The transfer line temperature was set at 250 °C, with a total run time of 42 min. Mass spectra were acquired in electron impact ionization mode (70 eV) with a scan range of m/z 50–550, a source temperature of 230 °C, and a quadrupole temperature of 150 °C.

Mass spectral identification was conducted by comparing the acquired spectra against reference data from the W8N08 mass spectral library (John Wiley & Sons, Inc., Hoboken, NJ, USA), and compounds were tentatively identified based on spectral similarity.

### 2.6. Statistical Analysis

The results obtained from the 2^2^ factorial design experiment were analyzed using Design Expert software (Version 12.0, Stat-Ease Inc., Minneapolis, MN, USA). Statistical analysis was performed using the SPSS software package (Version 17.0, SPSS Inc., Chicago, IL, USA). Group differences were assessed using one-way analysis of variance (ANOVA), and mean comparisons were subsequently performed using Tukey’s post hoc test. A significance level of *p* < 0.05 was applied for all statistical analyses. Principal component analysis (PCA) biplots generated by SPSS (Version 17.0) were used to illustrate the relationships between treatments and their associated chemical properties.

## 3. Results and Discussion

### 3.1. Influence of Drying Conditions on the Physicochemical Properties, Umami Composition and Sensory Evaluation of D-HBb

#### 3.1.1. Physicochemical Properties and Umami Composition of D-HBb

The physicochemical properties and umami composition responses of D-HBb under different drying conditions, including moisture content, color values, aw, protein contents, 5′-nucleotide compounds, and EUC, are presented in Table 2. The physicochemical properties, amino acid composition, 5′-nucleotide content, and EUC values reported in Table 2 and Table 3 refer to dried honeybee brood (D-HBb) obtained directly from the drying treatments. In contrast, HBb-UP refers to the foam-mat dried umami powder prepared from the optimized extract and subsequently used for volatile, sensory, and electronic tongue analyses. The moisture content of D-HBb ranged from 2.17% to 53.31%, while aw values varied between 0.33 and 0.94. An increase in drying temperature and time resulted in decreased moisture content and aw. These findings are consistent with the study of Ikhsan et al. [21], who reported that higher temperatures and prolonged drying time reduce moisture content and aw.

D-HBb samples were evaluated for color parameters L*, a*, and b*, representing lightness, redness, and yellowness, respectively. The L*, a*, and b* values ranged from 54.69 to 63.20, 5.21 to 11.23, and 11.46 to 18.01, respectively. Overall, changes in drying temperature and time did not significantly affect the color parameters (L*, a*, and b*) of D-HBb. Although slight differences were observed among the experimental runs, these variations were small and did not indicate noticeable color deterioration. Furthermore, the protein content of D-HBb samples was analyzed for each experimental run, with values ranging from 35.60 to 52.22 g protein per 100 g sample. In this study, the effect of the drying process on the levels of 5′-GMP, 5′-IMP, and 5′-XMP in D-HBb was investigated. The results showed that the highest level of 5′-GMP was found at 50 °C for 24 h, reaching approximately 81.82 mg/100 g db. On the other hand, the highest level of 5′-IMP was observed at 80 °C for 24 h, reaching approximately 27.51 mg/100 g db. Additionally, the highest level of 5′-XMP was found at a drying temperature of 80 °C for 3 h, reaching approximately 289.27 mg/100 g db. Shen et al. [22] similarly found that drying conditions substantially influence the concentrations of free amino acids and 5′-nucleotides, both essential for umami flavor development. Their research showed that elevating drying temperatures increased these compounds until reaching an optimal point, after which thermal degradation reduced their levels. Findings reported by Wang et al. [23] indicated that during thermal drying, the total content of 5′-nucleotides initially increased and subsequently decreased with rising temperature, indicating that temperature plays a critical role in regulating 5′-nucleotide levels. The EUC, which represents umami taste intensity, ranged from 13.96 to 80.58 g MSG/100 g db. The maximum EUC value of approximately 80.58 g MSG/100 g db was achieved at 50 °C after 24 h. Previous findings by Yang et al. [24] indicated that proper thermal treatment may enhance EUC values in food products. Drying at higher temperatures can increase free amino acid concentrations, particularly glutamic acid and aspartic acid, which contribute to umami taste [25]. The results indicated that all drying conditions exhibited a statistically significant increase in EUC and 5′-GMP values with increasing drying temperature and time. A similar trend was reported by Hou et al. [25], who observed that hot-air drying altered umami-related compounds in *Suillus granulatus*, supporting the increase in EUC and 5′-GMP values observed at higher drying temperatures and longer drying durations in the present study, with samples dried at 60 °C exhibiting markedly higher EUC, glutamic acid, and 5′-GMP levels. The authors suggested that moderate thermal processing promotes the degradation of intracellular RNA into 5′-nucleotides through endogenous enzymatic activity before enzyme inactivation occurs. Additionally, moisture removal during drying may concentrate umami-related compounds and enhance their synergistic interaction, thereby increasing EUC values. However, excessively high temperatures could potentially lead to nucleotide degradation, indicating that both temperature and drying time play critical roles in modulating umami intensity. These findings support the conclusion that increasing drying temperature and time, within an appropriate range, significantly enhances EUC and 5′-GMP contents.

The amino acid compositions of D-HBb from each experimental run are presented in Table 3. The total amino acid concentration ranged from 482.71 to 906.71 mg/100 g db, with the highest value (approximately 906.71 mg/100 g db) observed at 65 °C for 13.5 h. The major amino acids identified across all experimental runs included phenylalanine, alanine + cysteine, glutamic acid, and proline. The phenylalanine concentration in D-HBb (*Apis mellifera* L.) contributes to its status as a highly sustainable and protein-rich food source, suitable for human consumption. These findings suggest potential applications of D-HBb as a protein-rich ingredient in functional food formulations [2,26,27]. Interestingly, glutamic acid concentrations ranged from 86.14 to 180.47 mg/100 g db, while aspartic acid ranged from 2.50−7.61 mg/100 g db. These amino acids play an important role in umami perception by interacting with receptors responsible for taste signaling [19]. The presence of umami-related amino acids, particularly glutamic and aspartic acids, supports the potential use of D-HBb as a natural flavor-enhancing ingredient. Additionally, the overall amino acid profile suggests its suitability for incorporation into value-added food products.

#### 3.1.2. Sensory Evaluation Responses Under Different Drying Conditions of D-HBb

The average sensory scores for appearance, color, aroma, and overall acceptance of D-HBb under different drying conditions are presented in Table 4. Based on the 9-point hedonic scale, the scores ranged from 3.10 to 7.30 for appearance, 4.10 to 7.20 for color, 3.90 to 6.80 for aroma, and 3.70 to 6.85 for overall acceptance. Overall, the sensory scores were within an acceptable range (approximately ≥ 5) across all experimental runs.

Drying temperature and time were associated with variations in sensory attributes, particularly in terms of appearance and aroma. Samples processed under moderate drying conditions generally achieved higher scores compared with those subjected to either low or excessively high temperatures. Moisture reduction combined with heat-related reactions during drying may have contributed to this observed trend. Moderate thermal treatment may promote desirable aroma development through controlled Maillard reactions and the formation of volatile compounds, whereas excessive drying may result in pigment degradation, surface darkening, and the formation of off-flavors, leading to reduced sensory acceptance.

A similar pattern was observed for aroma scores, suggesting that drying conditions influence the development of volatile compounds. Thermal processing may facilitate the release and transformation of flavor precursors, including amino acids and nucleotides, which contribute to savory characteristics. However, excessive temperature or prolonged drying time may lead to the degradation or loss of key aroma compounds, negatively affecting sensory evaluation.

Overall acceptance reflected the combined influence of appearance, color, and aroma, indicating that consumer preference was dependent on drying conditions. These findings are consistent with the observed variations in umami-related compounds and volatile profiles, suggesting a relationship between chemical composition and sensory evaluation.

#### 3.1.3. Influence of Drying Temperature and Time on the Physicochemical Properties and Umami Composition of D-HBb

The analysis of variance (ANOVA, *p* < 0.01), including the fitted regression models, adjusted R^2^, and *p*-value, for the selected dependent variables of D-HBb is presented in Table 5. Moisture content, aw, 5′-GMP, EUC values, and appearance of D-HBb were adequately described by a two-factor interaction (2FI) model. In contrast, aroma and overall acceptance were better fitted to a quadratic model. Drying temperature and time were significantly associated with variations in these parameters. These results indicate that drying conditions primarily influenced moisture-related and umami-related parameters.

**Figure 1:**
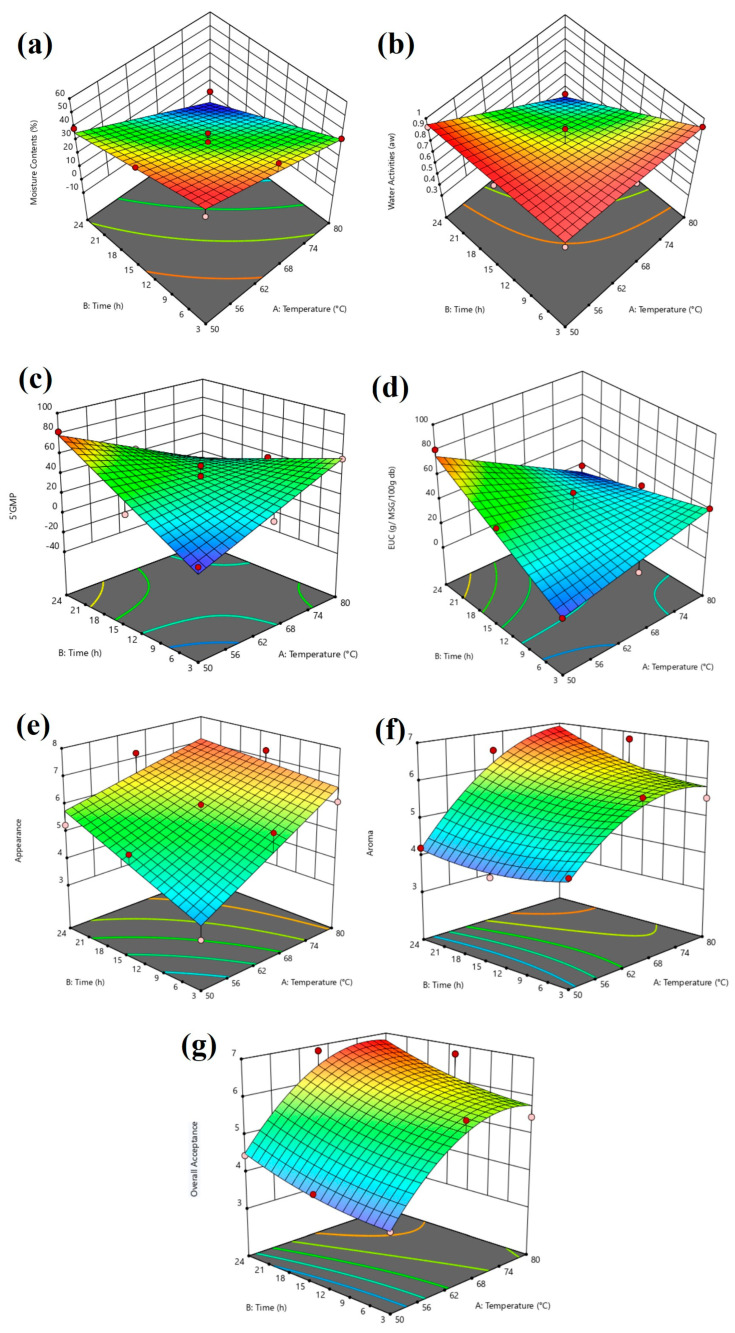
Three-dimensional (3D) surface and contour plots illustrating the interaction effects of the drying temperature and time on the physicochemical properties and umami composition of D-HBb, including (**a**) moisture content, (**b**) aw, (**c**) 5′-GMP, (**d**) EUC, (**e**) appearance, (**f**) aroma, and (**g**) overall acceptance.

Drying temperature and time showed a negative correlation with moisture content and aw, both of which decreased as temperature and drying time increased. Simultaneously, increases in drying temperature and time enhanced 5′-GMP content, as well as appearance, aroma, and overall acceptance. Drying time had a significant effect on EUC, whereas temperature exerted a comparatively smaller influence, as illustrated in Figure 1. Longer drying time resulted in higher EUC values, whereas temperature exhibited a less marked effect on umami concentration. This trend suggests that drying time is an important factor in modulating umami intensity. Comparable results were reported by Abasi et al. [28], who found that both moisture content and aw declined as drying temperature and time increased, while moisture changes became less evident during the later phase of the drying process. Low moisture content is closely associated with low aw, as reduced moisture limits the availability of free water for chemical reactions [29]. The enhancement of 5′-GMP content was strongly influenced by both drying temperature and time. Sombutyanuchit et al. [30] reported that optimal drying conditions promoted 5′-GMP accumulation by regulating enzyme activity and preserving nucleotide stability. Although elevated temperatures accelerated nucleotide conversion, excessive heat led to nucleotide degradation. Similarly, Mior Zakuan Azmi et al. [31] demonstrated that extended baking times combined with higher temperatures altered product texture and sensory evaluation, often improving consumer acceptance depending on the specific processing conditions. However, excessively high temperatures may compromise nutritional quality or flavor attributes [32].

#### 3.1.4. Optimization of Drying Temperature and Time for D-HBb Production

Response surface methodology (RSM) was applied to determine the optimal drying conditions for D-HBb, with the objective of minimizing moisture content and aw while maximizing 5′-GMP and EUC. In addition, sensory attributes, including appearance, aroma, and overall acceptance, were constrained within the experimentally observed ranges. The optimal conditions were determined by numerical optimization using Design-Expert^®^ software, which identified 65 °C for 3 h as the optimal drying parameters. Under these conditions, a desirability value of 0.675 was obtained, with predicted 5′-GMP and EUC values of 45.75 mg/100 g db and 44.33 g MSG/100 g db, respectively. The predicted physicochemical properties of D-HBb included a moisture content of 14.69% and an aw value of 0.63. Moreover, the predicted sensory scores for appearance, aroma, and overall acceptance were 6.46, 6.17, and 6.45, respectively.

Although the highest EUC value was obtained at 50 °C for 24 h, numerical optimization identified 65 °C for 3 h as the most desirable condition when multiple responses were considered simultaneously. While prolonged drying at a lower temperature may enhance the accumulation of certain umami-related compounds, it also reduces processing efficiency and may lead to quality deterioration due to extended thermal exposure. In contrast, the optimized condition maintained relatively high levels of umami-active compounds while providing acceptable sensory quality and favorable physicochemical properties. The predicted moisture content (14.69%) and water activity (aw = 0.63) suggest improved storage stability and may help limit microbial growth and quality deterioration during storage. Furthermore, the substantially shorter drying time offers practical advantages in terms of process efficiency and energy utilization. Therefore, the selected condition represents a balanced compromise between flavor quality, product stability, and manufacturing feasibility, supporting its suitability for HBb-UP production.

### 3.2. Comparison of Gas Chromatography–Mass Spectrometry (GC-MS) and Electronic Nose (E-Nose) Analyses of Volatile Compounds in Optimized HBb-Up

**Figure 2:**
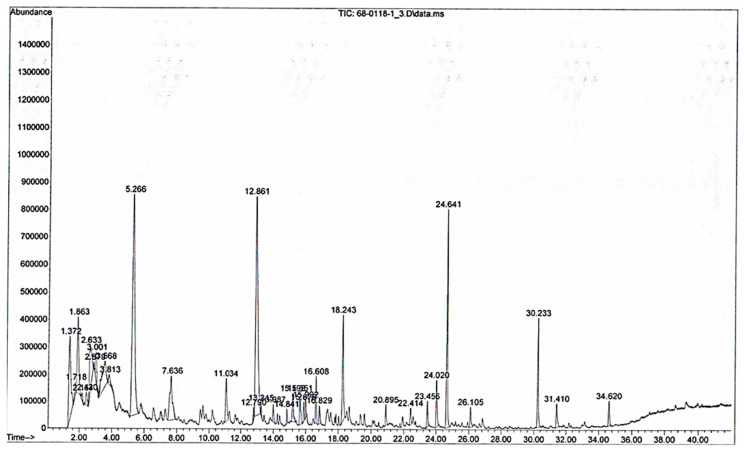
Chromatographic profiles of volatile compounds in optimized HBb-UP obtained by GC–MS.

GC–MS characterization of the optimized HBb-UP sample revealed the presence of 34 volatile compounds (Figure 2), including hydrocarbons, aldehydes, terpenes, alcohols, pyrazines, furans/lactones, and sulfur- and nitrogen-containing heterocycles. Among these, hydrocarbons and terpenes were the predominant groups. The major compounds detected were hexane, limonene, nonanal, 2-ethyl-1-hexanol, and 4-hydroxy-2,5-dimethyl-3(2H)-furanone, which are commonly associated with fatty, citrus, floral, green, and sweet caramel-like aroma notes.

**Figure 3:**
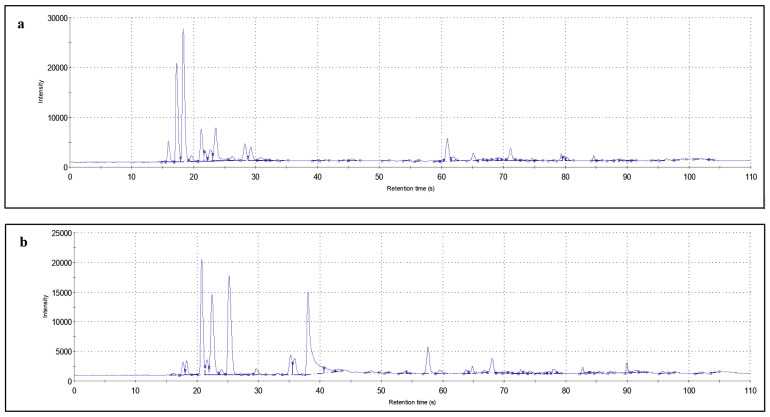
Chromatographic profiles of volatile compounds in optimized HBb-UP obtained by Electronic Nose using MXT-5 (**a**) and MXT-1701 columns (**b**).

Electronic nose (e-nose) analysis revealed volatile profiles corresponding to 36 compounds in the optimized HBb-UP sample (Figure 3). The chromatographic profiles of these volatile compounds were obtained using two different capillary columns: MXT-5 (Figure 3a) and MXT-1701 (Figure 3b). The MXT-5 column (non-polar) primarily separates compounds based on volatility and boiling point, whereas the MXT-1701 column (mid-polar) provides enhanced separation of polar volatile compounds. The use of both columns allows for improved resolution and more comprehensive identification of aroma-related compounds in the optimized HBb-UP sample. The predominant groups detected included alcohols, aldehydes, ketones, terpenes, furans, and lactones, with alcohols and aldehydes being the most prominent. Major detected compounds included methanol, ethanol, nonanal, limonene, and 4-hydroxy-5-methyl-3(2H)-furanone, corresponding to alcoholic, green–fatty, citrus, fruity, and caramel-like aroma notes. The detected volatile compounds may originate from multiple pathways associated with both intrinsic honeybee brood composition and thermal processing. Aldehydes such as nonanal are commonly associated with lipid oxidation of unsaturated fatty acids, whereas furan compounds, including 4-hydroxy-5-methyl-3(2H)-furanone, are often linked to Maillard-related reactions during heating, contributing caramel-like aroma characteristics [33,34,35]. Alcohols and terpenes may partially reflect naturally occurring brood-derived aroma constituents, while thermal processing may further influence their formation and release. These classifications are proposed as plausible interpretations based on previously reported food flavor chemistry.

Both analytical approaches revealed a complex volatile profile with several overlapping compounds, such as nonanal, limonene, and furanone derivatives, confirming their importance in aroma development of optimized HBb-UP. Key volatile compounds with desirable odor attributes are summarized in Table 6, including fruity, citrus, floral, green, nutty, roasted, caramel-like, and creamy notes, which collectively contribute to the overall aroma complexity. Notably, the E-nose detected a slightly higher number of volatile signals compared to GC–MS. This difference was attributed to the higher sensitivity of the E-nose sensor array to low-molecular-weight volatiles and its ability to capture a broader range of compounds, including those present in trace amounts that may not have been efficiently separated or identified by GC–MS [36]. According to a previous study by Haber et al. [10], GC–MS analysis with HS-SPME injection of honeybee brood (larvae and pupae) identified several key odor-active compounds, including 2- and 3-methylbutanal, diacetyl, nonanal, dimethyl sulfide, and ocimene, which were strongly associated with buttery and milky aroma attributes. Moreover, 3-methylbutanal (isovaleraldehyde) and nonanal were also detected in this study, thereby reinforcing their significance as key odor-active compounds contributing to the characteristic aroma profile of optimized HBb-UP.

### 3.3. Evaluation of Optimized HBb-Up Using Descriptive Analysis (DA) and Electronic Tongue (E-Tongue)

**Figure 4:**
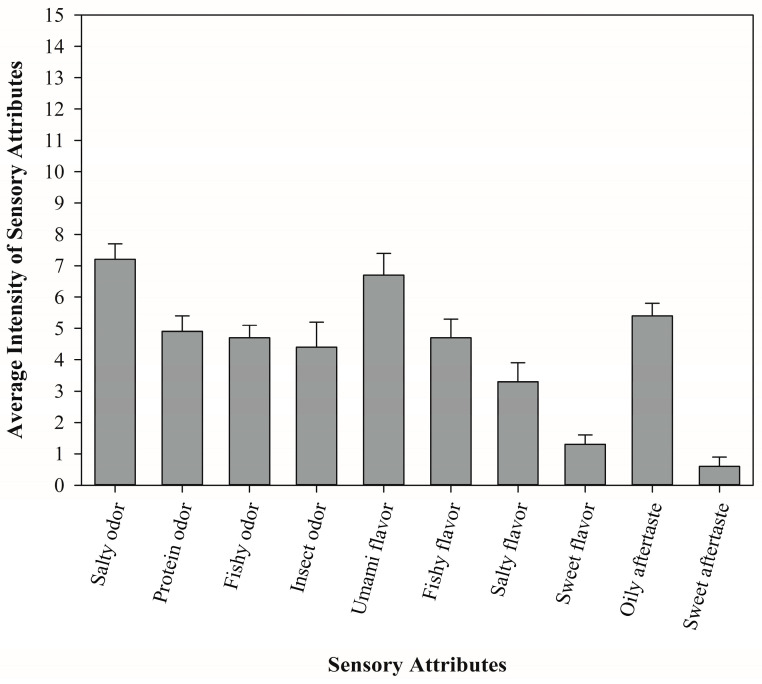
Sensory attribute intensities of optimized HBb-UP evaluated by Descriptive Analysis (DA).

**Figure 5:**
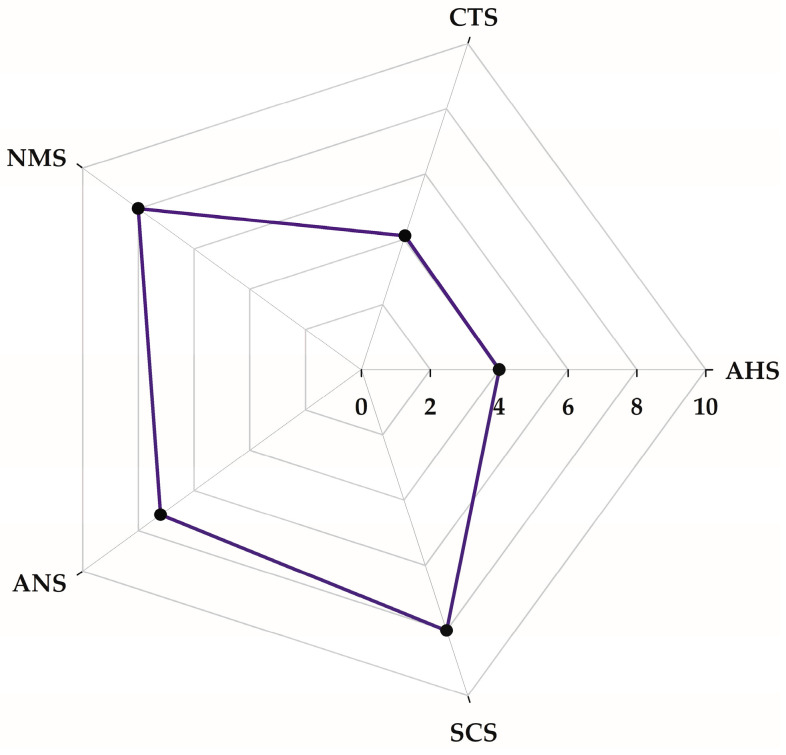
Radar chart of Electronic Tongue (E-Tongue) Analysis of the Taste Profile of optimized HBb-UP. AHS, CTS, NMS, ANS, and SCS represent the basic human taste perceptions, where AHS corresponds to sourness, CTS to saltiness, NMS to umami, ANS to sweetness, and SCS to bitterness.

Optimized HBb-UP was evaluated using descriptive analysis (DA), comprising 16 attributes scored on a 15-point intensity scale. The sensory attribute intensities of the optimized sample are shown in Figure 4 and included odor, flavor, and aftertaste attributes. The results indicated that odor attributes were characterized by salty odor (7.2 ± 0.5), protein odor (4.9 ± 0.5), fishy odor (4.7 ± 0.4), and insect odor (4.4 ± 0.8). Flavor perceptions included umami flavor (6.7 ± 0.7), fishy flavor (4.7 ± 0.6), salty flavor (3.3 ± 0.6), and sweet flavor (1.3 ± 0.3). Aftertaste attributes were characterized by oily aftertaste (5.4 ± 0.4) and sweet aftertaste (0.6 ± 0.3). These results indicate that optimized HBb-UP exhibited a complex sensory profile dominated by umami-related and fatty attributes. The taste profile of optimized HBb-UP was further evaluated using an electronic tongue (E-tongue), as shown in Figure 5. The electronic tongue provides comparative taste pattern recognition based on combined sensor responses rather than direct quantification of individual taste compounds, allowing discrimination of taste characteristics among samples. A moderate response signal (3.6 ± 0.4) was observed, and the dominant taste attributes included umami (8.0), bitterness (8.0), sweetness (7.2), saltiness (4.1), and sourness (4.0). These instrumental results were generally consistent with the DA findings, particularly in confirming the prominence of umami taste. Although umami was identified as a dominant taste attribute, the E-tongue analysis also revealed a relatively high bitterness response (8.0), comparable to the umami signal. This bitterness may be associated with amino acids, peptides, or thermally generated compounds formed during processing. Nevertheless, the descriptive sensory analysis did not indicate dominant bitterness perception, suggesting that interactions among umami, saltiness, and other taste modalities may contribute to taste balance and overall flavor acceptability of optimized HBb-UP. The comparable responses obtained from DA and the E-tongue further support the involvement of umami-related compounds in the perceived taste profile, including glutamic acid, aspartic acid, and 5′-nucleotides, as well as EUC values, thus contributing to the overall taste perception of optimized HBb-UP. This consistency highlights the relationship between chemical composition and sensory perception.

Although the E-Tongue is limited to specific taste modalities, it demonstrated a strong ability to objectively detect and quantify the principal taste characteristics of optimized HBb-UP with high reproducibility and sensitivity. These findings indicate that DA provides a more comprehensive evaluation of complex sensory attributes across multiple modalities through human perception, whereas the E-tongue offers precise, rapid, and unbiased measurements of key taste attributes [38]. Collectively, the combined application of DA and the E-tongue highlights their complementary strengths by integrating human sensory perception with instrumental accuracy, thereby enabling a more comprehensive and reliable characterization of the sensory profile of optimized HBb-UP.

### 3.4. Correlation Between Physicochemical Properties, Umami Composition, and Descriptive Analysis of Optimized HBb-Up

**Figure 6:**
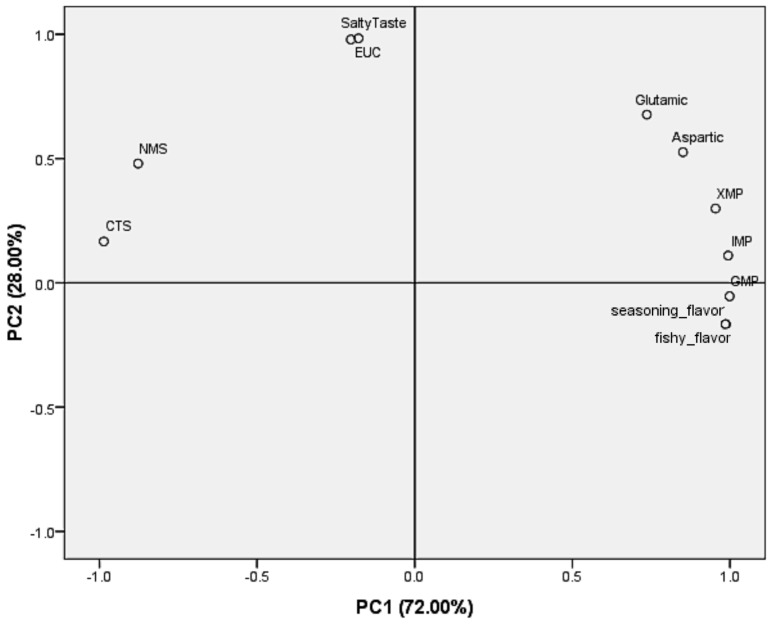
Principal component analysis (PCA) illustrating synergistic correlations between umami-related compounds and sensory attributes of optimized HBb-UP.

Principal component analysis (PCA) illustrating the relationships between umami-related compounds and sensory attributes of optimized HBb-UP is shown in Figure 6. The PCA biplot indicates distinct associations among taste-related variables. Variables located in close proximity exhibit similar loading patterns, suggesting their contribution to related sensory evaluation. Glutamic acid, aspartic acid, 5′-IMP, 5′-GMP, and 5′-XMP are tightly clustered on the positive side of PC1, indicating a strong correlation between amino acids and 5′-nucleotides that collectively enhance EUC. The EUC value is widely applied to assess umami intensity in food systems, as it represents the synergistic interaction between umami-related free amino acids and taste-enhancing nucleotides [39]. Their proximity to the descriptors seasoning flavor and fishy flavor further suggests synergistic interactions among these compounds in contributing to umami-rich and complex flavor characteristics. Similarly, in the upper region of the biplot, EUC and salty taste appear in proximity, indicating a positive association between saltiness and umami enhancement. The observed association between EUC and saltiness may be related to synergistic interactions between umami compounds and salt taste perception. Previous studies have suggested that umami substances, particularly glutamic acid and 5′-nucleotides, can enhance saltiness perception through taste receptor crosstalk and modulation of saliva secretion, thereby increasing flavor intensity and mouthfeel [39,40]. This interaction may contribute to improved palatability while allowing sodium reduction, supporting the potential application of HBb-UP as a natural flavor enhancer in low-sodium food formulations [38,41]. The variables CTS (saltiness) and NMS (umami), identified by the E-tongue analysis, are also located in this quadrant, supporting their roles in enhancing the perceived saltiness and umami intensity of optimized HBb-UP.

These findings are consistent with previous studies. Li et al. [39] reported that elevated EUC values correlated with stronger saltiness perception, suggesting a synergistic interaction between umami-active compounds (such as glutamic acid, aspartic acid, and 5′-nucleotides) and salt. This synergism enhances overall flavor intensity without the need for additional sodium, providing both sensory and nutritional benefits. These findings suggest that umami substances not only contribute to flavor depth and complexity but also act as natural saltiness enhancers, representing a promising strategy for the development of reduced-sodium foods while maintaining sensory appeal.

The positive association observed between saltiness and umami in the PCA analysis of optimized HBb-UP aligns with previous findings by Song et al. [42], who reported a strong positive correlation between these two taste modalities. Their study indicated that an increase in umami intensity enhances the perception of saltiness, emphasizing the interactive relationship between umami and saltiness in complex food matrices. The association observed may result from interactions between amino acids, particularly glutamic and aspartic acids, and flavor-enhancing nucleotides such as AMP, IMP, and GMP, which together contribute to overall taste perception. Consequently, umami-active compounds may serve as potential natural saltiness enhancers, providing both sensory and functional benefits for flavor optimization in reduced-sodium and seasoning-type food products.

## 4. Conclusions

This study evaluated the influence of temperature and time on the physicochemical properties and sensory evaluation of honeybee brood. The 2^2^ factorial design revealed that drying conditions were associated with variations in 5′-nucleotide formation, EUC, and umami-related amino acids, particularly glutamic and aspartic acids. The optimized drying conditions (65 °C for 3 h) improved the overall physicochemical properties and sensory evaluation of honeybee brood umami powder (HBb-UP). Gas Chromatography–Mass Spectrometry (GC-MS) and electronic nose analyses confirmed the presence of key volatile compounds, including nonanal, limonene, and furanone derivatives, which contributed to the characteristic aroma profile of optimized HBb-UP. Descriptive sensory evaluation and electronic tongue responses further demonstrated that umami and saltiness predominated in the taste profile. Principal component analysis (PCA) results revealed strong positive correlations among amino acids, nucleotides, EUC, and sensory descriptors, particularly highlighting the synergistic relationship between umami-related compounds and taste perception. Overall, these findings indicate that umami-active compounds contribute to the flavor complexity of optimized HBb-UP. In addition to dominant umami and salty notes, optimized HBb-UP exhibited seasoning-like and mild fishy attributes associated with amino acid–nucleotide interactions and volatile compounds. These integrated flavor characteristics define the distinctive sensory profile of optimized HBb-UP and suggest its potential as a functional ingredient for flavor enhancement in food applications derived from edible insects.

## Figures and Tables

**Table 1:** Experimental conditions of the 2^2^ factorial design with center-point replicates for honeybee brood drying optimization.

Level Code	Variables and Factor Level	
	Temperature (°C)	Time (h)
−	50	3
0	65	13.50
+	80	24
**Run**	**Temperature**	**Time**
1	−	−
2	+	−
3	−	+
4	+	+
5	−	0
6	+	0
7	0	−
8	0	+
9	0	0
10	0	0

Values are expressed as coded levels: − indicates the low level; 0 indicates the center level; + indicates the high level of each variable.

**Table 2:** Physicochemical properties and umami composition responses of D-HBb under different drying conditions.

Run	Responses									
	Moisture Contents (%)	Color Values			aw	5′-Nucleotide Compounds (mg/100 g db)			Protein Contents (g/100 g db)	EUC (g MSG/100 g db)
		L*	a*	b*		5′-GMP	5′-IMP	5′-XMP		
**1**	53.31 ± 2.60	62.78 ± 0.06	5.46 ± 0.07	12.24 ± 0.05	0.93 ± 0.01	3.91 ± 0.25	14.17 ± 0.14	162.45 ± 0.16	35.60 ± 0.89	13.96 ± 0.19
**2**	34.23 ± 4.78	58.48 ± 0.55	6.10 ± 0.08	11.73 ± 0.58	0.93 ± 0.02	56.51 ± 0.89	18.52 ± 0.34	289.27 ± 0.80	39.29 ± 0.94	41.60 ± 0.68
**3**	39.15 ± 4.16	62.18 ± 1.20	6.14 ± 0.06	15.66 ± 0.40	0.93 ± 0.01	81.82 ± 4.90	10.08 ± 0.03	278.16 ± 0.96	38.62 ± 0.52	80.58 ± 1.96
**4**	2.17 ± 0.07	58.51 ± 1.46	11.23 ± 0.03	15.55 ± 0.26	0.33 ± 0.05	9.35 ± 0.34	27.51 ± 0.50	168.16 ± 0.23	36.81 ± 0.04	18.88 ± 0.35
**5**	48.03 ± 0.42	60.08 ± 0.89	5.21 ± 0.10	12.49 ± 0.21	0.92 ± 0.01	25.11 ± 3.10	21.92 ± 0.20	243.53 ± 0.35	48.86 ± 0.73	49.25 ± 1.22
**6**	3.59 ± 0.08	60.01 ± 0.04	5.61 ± 0.23	11.46 ± 1.82	0.49 ± 0.02	36.16 ± 0.41	20.62 ± 0.63	176.64 ± 2.07	38.19 ± 0.28	30.36 ± 1.04
**7**	52.18 ± 3.53	63.20 ± 0.03	9.26 ± 0.22	18.01 ± 1.03	0.94 ± 0.01	19.38 ± 2.26	8.66 ± 0.01	183.13 ± 0.51	52.22 ± 0.35	18.44 ± 0.92
**8**	4.38 ± 0.38	60.45 ± 0.16	7.47 ± 0.47	15.74 ± 1.18	0.58 ± 0.01	44.27 ± 2.83	13.38 ± 0.19	202.72 ± 1.26	39.08 ± 0.69	25.01 ± 1.43
**9**	37.09 ± 2.31	54.69 ± 0.57	6.40 ± 0.66	14.17 ± 0.10	0.92 ± 0.01	49.12 ± 2.59	17.42 ± 0.36	265.20 ± 0.19	40.19 ± 1.43	50.49 ± 1.05
**10**	30.56 ± 4.24	55.00 ± 1.03	6.50 ± 0.32	12.70 ± 0.15	0.91 ± 0.02	38.66 ± 2.58	13.19 ± 0.37	235.01 ± 0.64	36.62 ± 2.06	29.98 ± 1.20

Values are expressed as mean ± standard deviation, *n* = 3, aw = water activity; 5′-GMP = Guanosine-5′-monophosphate; 5′-IMP = Inosine-5′-monophosphate; 5′-XMP = Xanthosine-5′-monophosphate, EUC = equivalent umami concentrations; db = dry weight basis; Moisture content is expressed on a wet basis (%), whereas protein content, amino acids, and 5′-nucleotides are expressed on a dry basis (g/100 g db or mg/100 g db, as applicable); D-HBb: dried honeybee brood obtained directly from drying treatments.

**Table 3:** Amino acid composition of D-HBb from each experimental run determined by HPLC.

Amino Acids (mg/100 g db)	Runs									
	1	2	3	4	5	6	7	8	9	10
**Aspartic acid**	3.88 ± 0.15	3.35 ± 0.14	3.88 ± 0.22	3.21 ± 0.28	6.61 ± 0.13	5.52 ± 0.11	2.50 ± 0.04	3.28 ± 0.12	7.61 ± 0.12	4.14 ± 0.01
**Threonine**	15.46 ± 0.21	13.53 ± 0.02	16.85 ± 0.55	ND	16.92 ± 0.20	19.19 ± 0.26	17.08 ± 0.35	11.04 ± 0.74	16.94 ± 0.03	12.69 ± 0.01
**Serine**	1.94 ± 0.08	14.39 ± 0.49	16.45 ± 0.07	15.36 ± 0.08	16.52 ± 0.01	5.86 ± 0.58	1.71 ± 0.06	4.30 ± 0.08	7.04 ± 0.21	5.22 ± 0.06
**Glutamic acid**	94.07 ± 2.21	105.53 ± 3.71	180.47 ± 0.26	102.61 ± 2.65	177.95 ± 0.58	117.94 ± 0.01	92.18 ± 0.92	86.14 ± 0.15	142.20 ± 0.87	100.60 ± 0.01
**Proline**	76.14 ± 0.35	73.22 ± 0.45	84.18 ± 0.26	59.31 ± 0.63	96.70 ± 0.25	60.81 ± 0.12	71.28 ± 0.53	53.28 ± 0.71	81.36 ± 0.49	65.95 ± 0.01
**Glycine**	13.53 ± 0.23	18.37 ± 0.20	22.22 ± 0.16	14.13 ± 0.19	25.44 ± 0.04	17.02 ± 0.08	13.85 ± 0.16	14.16 ± 0.09	23.01 ± 0.35	17.55 ± 0.03
**Alanine +** **Cystine**	90.07 ± 2.66	117.93 ± 1.45	129.40 ± 8.04	276.60 ± 2.61	176.39 ± 0.98	181.98 ± 2.80	83.84 ± 3.65	108.61 ± 0.01	253.06 ± 5.47	156.47 ± 0.01
**Valine**	13.19 ± 1.61	18.68 ± 0.45	20.72 ± 1.39	19.74 ± 0.71	23.81 ± 0.33	21.25 ± 0.14	13.74 ± 5.50	14.30 ± 0.64	23.69 ± 0.18	16.92 ± 0.01
**Methionine**	1.32 ± 1.01	1.52 ± 0.11	2.89 ± 1.20	1.63 ± 0.01	3.03 ± 0.29	1.86 ± 0.49	2.01 ± 3.84	1.10 ± 0.27	1.83 ± 0.04	1.26 ± 0.05
**Isoleucine**	2.39 ± 0.21	5.58 ± 0.19	5.17 ± 0.15	5.93 ± 0.34	6.07 ± 0.01	6.38 ± 0.18	2.84 ± 0.12	4.46 ± 0.12	7.52 ± 0.14	5.52 ± 0.01
**Leucine**	5.92 ± 0.01	7.90 ± 0.17	8.19 ± 0.45	9.15 ± 0.18	10.41 ± 1.23	9.46 ± 0.38	5.36 ± 0.07	6.48 ± 0.65	11.11 ± 0.06	8.14 ± 0.02
**Tyrosine**	ND	7.98 ± 0.24	4.71 ± 0.07	5.48 ± 0.29	15.10 ± 0.39	14.79 ± 0.13	ND	6.46 ± 2.31	10.47 ± 3.58	7.92 ± 0.01
**Phenyl-** **alanine**	152.70 ± 2.02	203.49 ± 3.52	248.88 ± 3.10	167.46 ± 1.27	263.07 ± 1.30	198.05 ± 0.02	149.63 ± 0.38	155.27 ± 4.23	251.03 ± 0.81	187.74 ± 0.03
**Histidine**	4.16 ± 1.06	5.93 ± 0.06	7.38 ± 0.17	12.96 ± 0.71	9.46 ± 1.46	8.13 ± 0.17	3.89 ± 1.07	5.29 ± 0.21	10.19 ± 0.92	6.59 ± 0.01
**Lysine**	23.18 ± 0.65	33.47 ± 1.06	36.91 ± 0.47	23.17 ± 0.52	41.52 ± 1.88	29.14 ± 0.75	22.80 ± 0.40	23.65 ± 0.58	36.66 ± 0.21	28.68 ± 0.01
**Arginine**	ND	20.77 ± 0.32	ND	16.44 ± 3.18	ND	20.64 ± 1.24	ND	14.57 ± 0.15	22.99 ± 1.92	15.50 ± 0.02
**TAAs**	497.95	651.64	788.30	733.18	889.00	718.02	482.71	512.39	906.71	640.89

Values are expressed as mean ± standard deviation, *n* = 3; ND = not detected; TAAs = Total amino acids, representing sum of 17 amino acids; db = dry weight basis; Alanine and cystine are reported as a combined value due to co-elution under the chromatographic conditions used; therefore, individual quantification was not possible; D-HBb: dried honeybee brood obtained directly from drying treatments.

**Table 4:** Sensory scores of each experimental run of D-HBb under different drying conditions using a 9-point hedonic scale.

Runs	Product Attributes			
	Appearance	Color	Aroma	Overall Acceptance
**1**	3.10 ± 1.62	4.10 ± 2.00	4.40 ± 1.88	3.70 ± 1.66
**2**	6.10 ± 1.52	6.15 ± 1.39	5.50 ± 1.73	5.45 ± 1.57
**3**	5.25 ± 1.41	5.80 ± 1.32	4.20 ± 1.32	4.45 ± 1.05
**4**	6.60 ± 1.14	6.55 ± 1.15	6.55 ± 1.19	6.45 ± 0.94
**5**	5.05 ± 1.50	5.65 ± 1.35	3.90 ± 1.33	4.00 ± 1.12
**6**	7.30 ± 0.80	7.05 ± 0.89	6.80 ± 1.40	6.75 ± 1.37
**7**	5.80 ± 1.06	6.10 ± 1.25	5.90 ± 1.29	5.85 ± 1.27
**8**	7.20 ± 0.77	7.20 ± 0.70	6.50 ± 1.32	6.85 ± 1.18
**9**	6.00 ± 1.26	6.10 ± 1.12	5.60 ± 1.27	5.65 ± 1.27
**10**	5.40 ± 1.31	5.70 ± 0.92	5.25 ± 1.29	5.25 ± 0.97

Values are expressed as mean ± standard deviation; *n* = 25; D-HBb: dried honeybee brood obtained directly from drying treatments.

**Table 5:** Equation models, adjusted R^2^, and *p*-value for the selected dependent variable responses of D-HBb.

Responses	Equations	Adjusted R^2^	*p*-Value
**Moisture Contents** **(%)** **Water Activities** **(aw)** **5′-GMP** **(mg/100 g db)** **EUC** **(g MSG/100 g db)**	=98.26 − 0.73(A) + 0.36(B) − 0.03(AB) =0.92 + 0.01(A) + 0.05(B) − 0.01(AB) =− 143.33 + 2.58(A) + 13.79(B) − 0.20(AB) =− 61.15 + 1.33(A) + 10.02(B) − 0.14(AB)	0.8343 0.8276 0.8588 0.6767	0.0028 0.0032 0.0018 0.0200
**Appearance** **Aroma** **Overall Acceptance**	=− 2.15 + 0.11(A) + 0.23(B) − 0.01(AB) =− 8.81 + 0.40(A) − 0.16(B) + 0.01(AB) − 0.01(A)^2^ + 0.01(B)^2^ =− 13.78 + 0.53(A) − 0.03(B) + 0.01(AB) − 0.01(A)^2^ + 0.01(B)^2^	0.7047 0.7479 0.7453	0.0154 0.0489 0.0498

A = Temperature (°C); B = Time (hours); D-HBb: dried honeybee brood obtained directly from drying treatments.

**Table 6:** Key volatile compounds with desirable aroma notes in optimized HBb-UP detected by both GC–MS and E-nose.

No.	Compound	Chemical Class	CAS No.	Aroma Description	
				Odor Type	Flavor Type
**1**	3-Methylbutanal (Isovaleraldehyde)	Aldehyde	590-86-3	Nutty, chocolate-like, malty	Roasted, nutty, malty
**2**	Pent-1-en-3-ol	Alcohol	616-25-1	Green, metallic, fresh	Green, fresh note
**3**	1-Hexanol	Alcohol	111-27-3	Grassy, floral, green	Green, floral
**4**	β-Pinene	Terpene	127-91-3	Woody, pine-like	Herbal, resinous
**5**	Limonene	Terpene	138-86-3	Citrus, lemon-like, fruity	Citrus, fruity
**6**	2-Ethyl-3,6-dimethylpyrazine	Pyrazine	13925-07-0	Roasted, earthy, nutty	Roasted, savory
**7**	Nonanal	Aldehyde	124-19-6	Fatty, citrus, green	Fresh, citrusy, green
**8**	4-Hydroxy-5-methyl-3(2H)-furanone	Furanone derivative	118-71-8	Cotton candy, caramel-like, sweet	Sweet, candy-like
**9**	*p*-Methylacetophenone	Ketone/Phenone	122-00-9	Floral, sweet, mild aromatic	Floral-sweet
**10**	Dodecane	Hydrocarbon (Alkane)	112-40-3	Waxy, faint oily	Neutral, background note
**11**	(Z)-Citral (Geranial isomer)	Aldehyde (Terpenoid)	141-27-5	Lemon, citrus, fresh	Sweet citrus
**12**	δ-Decalactone	Lactone	705-86-2	Fruity, peach-like, creamy	Fruity, creamy, peach-like

Aroma descriptions were sourced from the Good Scents Company Information System [37]. Selected volatile compounds detected by both GC–MS and electronic nose analyses are presented based on their relevance to the characteristic aroma profile of optimized HBb-UP. The complete volatile profile, including relative abundances of all detected compounds, is provided in Supplementary Table S2.

## Data Availability

The original contributions presented in this study are included in the article/Supplementary Material. Further inquiries can be directed to the corresponding authors.

## Supplementary Materials

The following supporting information can be downloaded at: https://www.mdpi.com/article/10.3390/foods15122234/s1, Table S1: Description of attributes and reference examples of optimized HBb-UP; Table S2: Volatile compounds and relative abundances detected in optimized HBb-UP by GC–MS and electronic nose analyses.

## Abbreviations

The following abbreviations are used in this manuscript:

a*	The red component
AHS	Sourness
ANOVA	Analysis of variance
ANS	Sweetness
aw	Water activity
b*	The yellow component
CTS	Saltiness
DA	Descriptive Analysis
D-HBb	Dried honeybee brood
E-tongue	Electronic tongue
EUC	Equivalent umami concentration
GC-E-Nose	Gas chromatograph–electronic nose
GC-MS	Gas chromatography–mass spectrometry
HBb-UP	Honeybee brood umami powder
L*	Brightness
NMS	Umami
SCS	Bitterness
