# Supplementary material for: Characterization of Umami Compounds and Volatile Profiles of Honeybee Brood Umami Powder Under Optimized Drying Conditions: Implications for Sensory Properties
Source: Foods. 2026 Jun 20;15(12):2234. doi: 10.3390/foods15122234 (PMC13297988; doi:10.3390/foods15122234)
Supplement: Supplementary file 1 [file foods-15-02234-s001.zip › foods-4358544-supplementary.pdf]

Supplementary material file

Characterization of Umami Compounds and Volatile Profiles of Honeybee Brood Umami Powder under Optimized Drying Conditions: Implications for Sensory Properties

Supakit Chaipoot [1,2,*], Sirinthip Jaijoi [1,3], Gochakorn Kanthakat [3], Kuntathee Chaimueng [1,3], Chalermkwan Somjai [4], Pairote Wiriyacharee [4], Rajnibhas Sukeaw Samakradhamrongthai [3], Pattavara Pathomrungsiyounggul [3], Worachai Wongwatcharayothin [5] and Rewat Phongphisutthinant [1,2,*]

[1] Multidisciplinary Research Institute, Chiang Mai University, Chiang Mai 50200, Thailand
[2] Center of Excellence in Microbial Diversity and Sustainable Utilization, Chiang Mai University, Chiang Mai 50200, Thailand
[3] Faculty of Agro-Industry, Chiang Mai University, Chiang Mai 50100, Thailand
[4] Processing and Product Development Factory, The Royal Project Foundation, Chiang Mai 50100, Thailand
[5] Faculty of Humanities, Chiang Mai University, Chiang Mai 50200, Thailand
* Correspondence: supakit.ch@cmu.ac.th; rewat.p@cmu.ac.th

Table S1. Description of attributes and reference examples of optimized HBb-UP.

Attributes	Descriptions	Reference examples
**Odor**		
Salty odor	Odor associated with salty or briny characteristics.	Seasoned shredded squid (Tao Thong brand), 1 g
Protein odor	Odor characteristic of protein-based materials.	Yeast extract solution (10 g in 150 mL water)
Fishy odor	Odor resembling fish or marine-like characteristics.	Matcha green tea solution (10 g in 150 mL water)
Insect odor	Odor characteristic of insect-derived materials.	Roasted honeycomb, 5 g and Fried black cricket, 3 g
**Flavor**		
Umami flavor	Flavor characteristic of seasoning sauces, typically associated with salty and umami notes.	Fa Thai seasoning powder (blue sachet) solution (3 g in 250 g water) and Fa Thai seasoning powder (blue sachet) solution (6 g in 250 g water)
Fishy flavor	Flavor resembling fish or marine-like characteristics.	Whey protein, ½ tsp and Fried silkworm, 1 tsp
Salty flavor	Basic flavor associated with salt (sodium chloride).	Sodium chloride solution (0.2%) and Sodium chloride solution (0.35%)
Sweet flavor	Basic flavor associated with sugars or sweet substances.	Sugar solution (2.0%)
**Aftertaste**		

Attributes	Descriptions	Reference examples
Oily aftertaste	Lingering oily sensation remaining in the mouth after swallowing.	Milk powder solution (50 g in 150 mL water)
Sweet aftertaste	Basic taste associated with sugars.	Sugar solution (2.0%)

The attribute terms and reference examples were established based on the consensus of the DA panelists during the training sessions.

Table S2. Volatile compounds and relative abundances detected in optimized HBb-UP by GC–MS and electronic nose analyses.

Peak No.	Gas Chromatography–Mass Spectrometry (GC-MS)		Peak No.	Electronic Nose (E-Nose) Analyses			
	Area (%)	Library/ ID		Area (%)	MXT-5 column	Area (%)	MXT-1701 column
1	5.96	1-Hexanol / Pent-1-en-3-ol	1	60.38	Unknown	156.64	Unknown
2	2.09	Unknown	2	1666.69	Methanol	894.04	Unknown
3	7.04	Unknown	3	7655.78	Ethanol	1016.40	Methanol
4	1.39	Unknown	4	17033.63	Propan-2-ol	83.06	Unknown
5	2.32	Unknown	5	610.46	Ethanethiol	8355.62	Ethanol
6	6.55	Unknown	6	3754.30	tert-Butyl methyl ether	1197.67	Unknown
7	1.82	Dodecane	7	1759.71	Butanal/ Hexane	8034.07	Propan-2-one
8	4.00	Dodecane	8	3952.19	2-Methylfuran	516.14	Ethanethiol
9	8.53	Dodecane	9	581.26	Methyl propanoate	1197.67	tert-Butyl methyl ether
10	4.55	Unknown	10	1783.90	(E)-But-2-enal/ 3-Methylbutanal	1197.67	Hexane
11	14.12	(Z)-Citral (Geranial isomer)/ Limonene/ β-Pinene	11	392.30	Pent-1-en-3-ol	81.96	Butanal
12	3.24	Unknown	12	92.04	Unknown	8304.07	2-Methylfuran
13	1.59	Nonanal	13	64.85	Unknown	516.14	Unknown
14	0.40	2-Ethyl-3,6-dimethylpyrazine	14	68.25	Unknown	10042.10	Unknown
15	13.15	Unknown	15	88.53	Unknown	81.96	Unknown
16	0.46	Unknown	16	165.77	Unknown	81.08	Unknown
17	0.90	1-Hexanol	17	162.57	Unknown	689.91	Methyl propanoate
18	0.29	Unknown	18	385.61	Unknown	193.61	Unknown
19	1.28	Unknown	19	52.87	1-Hexanol	1842.16	(E)-But-2-enal/ 3-Methylbutanal
20	1.00	Unknown	20	75.04	Unknown	274.39	Pent-1-en-3-ol
21	0.62	Unknown	21	112.34	Unknown	26.34	1-Hexanol
22	0.87	Unknown	22	50.62	Unknown	2532.73	β-Pinene
23	1.03	Unknown	23	2390.77	β-Pinene	492.23	Limonene
24	0.63	δ-Decalactone	24		Unknown	201.31	2-Ethyl-3,6-dimethylpyrazine
25	3.16	3-Methylbutanal (Isovaleraldehyde)	25	748.33	Limonene	230.15	n-Nonanal
26	0.55	4-Hydroxy-5-methyl-3(2H)-furanone	26	748.33	4-Hydroxy-2,5-dimethyl-3(2H)-furanone	197.75	Dodecane
27	0.50	Unknown	27	155.60	2-Ethyl-3,6-dimethylpyrazine	136.64	Unknown
28	0.71	Methane	28	127.91	2,4-Hexadienal	155.88	2,4-Hexadienoic acid

Peak No.	Gas Chromatography–Mass Spectrometry (GC-MS)		Peak No.	Electronic Nose (E-Nose) Analyses			
	**Area (%)**	**Library/ ID**		**Area (%)**	**MXT-5 column**	**Area (%)**	**MXT-1701 column**
29	1.49	Unknown	29	297.40	n-Nonanal	104.36	4-Hydroxy-5-methyl-3(2H)-furanone
30	5.12	*p*-Methylacetophenone	30	1347.80	Maltol	104.36	Maltol
31	0.55	5-Methyldihydro-2(3H)-furanone	31	101.00	*p*-Methylacetophenone	104.36	*p*-Methylacetophenone
32	2.63	Unknown	32	180.14	Dodecane	59.50	(Z)-Citral
33	0.75	Unknown	33	56.27	(Z)-Citral	534.60	Indole
34	0.72	2-Dimethylamino-6-hydroxypurine	34	371.84	Indole	50.74	δ-Decalactone
35	-	-	35	88.65	δ-Decalactone	51.02	Unknown
36	-	-	36	145.07	Unknown	77.37	Unknown
